# Echocardiographic Assessment of Embryonic and Fetal Mouse Heart Development: A Focus on Haemodynamics and Morphology

**DOI:** 10.1155/2014/531324

**Published:** 2014-02-23

**Authors:** Nathan D. Hahurij, Emmeline E. Calkoen, Monique R. M. Jongbloed, Arno A. W. Roest, Adriana C. Gittenberger-de Groot, Robert E. Poelmann, Marco C. De Ruiter, Conny J. van Munsteren, Paul Steendijk, Nico A. Blom

**Affiliations:** ^1^Department of Pediatric Cardiology, Leiden University Medical Center (LUMC), Leiden, P.O. Box 9600, 2300 RC, Leiden, The Netherlands; ^2^Department of Anatomy & Embryology, Leiden University Medical Center, P.O. Box 9600, 2300 RC, Leiden, The Netherlands; ^3^Department of Cardiology, Leiden University Medical Center, P.O. Box 9600, 2300 RC, Leiden, The Netherlands

## Abstract

*Background*. Heart development is a complex process, and abnormal development may result in congenital heart disease (CHD). Currently, studies on animal models mainly focus on cardiac morphology and the availability of hemodynamic data, especially of the right heart half, is limited. Here we aimed to assess the morphological and hemodynamic parameters of normal developing mouse embryos/fetuses by using a high-frequency ultrasound system. *Methods*. A timed breeding program was initiated with a WT mouse line (Swiss/129Sv background). All recordings were performed transabdominally, in isoflurane sedated pregnant mice, in hearts of sequential developmental stages: 12.5, 14.5, and 17.5 days after conception (*n* = 105). *Results*. Along development the heart rate increased significantly from 125 ± 9.5 to 219 ± 8.3 beats per minute. Reliable flow measurements could be performed across the developing mitral and tricuspid valves and outflow tract. M-mode measurements could be obtained of all cardiac compartments. An overall increase of cardiac systolic and diastolic function with embryonic/fetal development was observed. *Conclusion*. High-frequency echocardiography is a promising and useful imaging modality for structural and hemodynamic analysis of embryonic/fetal mouse hearts.

## 1. Introduction

Heart development is a complex process during which the heart will form from a single myocardial heart tube to a fully septated four-chambered heart with functional atrioventricular (AV) and ventriculoarterial valves and a separated outflow tract. The primary heart tube is derived from the splanchnic mesoderm in the embryonic plate and initially consists mainly of a left ventricle (LV) and AV canal. During further development, significant contributions to the heart tube will continue to be made from the mesenchyme situated behind the heart, the so-called second heart field (SHF). The cells of the SHF will form the right ventricle (RV) and outflow tract at the arterial pole of the heart as well as myocardial and vascular structures at the venous pole of the heart [1].

Abnormal heart development results in congenital heart disease (CHD), the most common birth defect with an estimated incidence of six per 1000 live born children [2]. In the last decade, knowledge regarding genes, growth and transcription factors important in heart development has dramatically increased, which has led to the generation of specific genetically mutated mouse models to study the etiology of CHD [3]. Up till now description of both normal and abnormal heart development in these mouse models mainly focused on the morphology of the heart, whereas functional data like hemodynamics and *in vivo* imaging are limited. Furthermore, functional parameters and their course at subsequent stages of normal mouse heart development have only been described in a few studies [4–7]. Specifically, knowledge regarding RV inflow and myocardial function during embryonic development is scarce.

In clinical practice ultrasound is the most frequently used minimally invasive imaging modality at pre- and postnatal stages of heart development. Clinical ultrasound machines have also been used for imaging of embryonic mouse hearts; however, ultrasound frequencies (8–15 MHz) are not sufficient for a detailed morphological and hemodynamic analysis. Recently developed high-frequency ultrasound systems (30–50 MHz) are able to offer sufficient spatial-temporal resolution for detailed and reliable imaging of embryonic mouse hearts. Thus far, there are only limited studies describing the hemodynamic and morphological changes at subsequent stages of heart development using such ultrasound systems [4, 6, 8].

Hemodynamics play an important role in normal heart development by the activation of shear-stress dependent signaling pathways. During development genes like Klf-2 [9] and periostin [10] are expressed in regions of the heart that are known to be subjected to high mechanical/hemodynamical stress. It has also been demonstrated that artificial alterations in hemodynamics throughout heart development result in aberrant activation of genes at the endocardial surface of the heart [11], which might lead to disturbed cardiogenesis, that is, CHD. A first step in the interpretation of hemodynamic parameters during development is the acquisition of a reliable reference series during normal heart development.

In the current study we aim to provide an overview of hemodynamic and morphological changes during embryonic and fetal stages of normal mouse heart development using a high-frequency ultrasound system. Knowledge of these functional parameters is highly relevant for the interpretation of results obtained in genetically mutated mouse models. We expect that knowledge of how echocardiographic parameters shift over development will eventually contribute to the early detection of CHD during human pregnancy.

## 2. Material and Methods

### 2.1. Animals

Animal experiments were approved by the local animal welfare committee (DEC number 10177) of the Leiden University Medical Center. A timed breeding program was initiated with a WT (Swiss/129Sv background) mouse line. The day after breeding was considered to be 0.5 days after conception (dpc). All pregnant mice (*n* = 11) were subjected to high-frequency ultrasound recordings at 3 subsequent stages of embryonic/fetal development, being 12.5, 14.5, and 17.5 dpc. At 12.5 dpc, the pregnant mother mouse was anaesthetized using isoflurane (induction 5%, maintenance during assessment 1.5%), the most commonly used anesthetic in mouse experiments [6, 12]. Subsequently, the sedated mouse was placed in the experimental setup (VisualSonics Vevo770 system, Toronto, Canada), which includes a heated table and rectal probe for maintenance and control of body temperature (36.5–37.5°C). Electrodes were attached to the paws for monitoring the maternal electrocardiogram and heart and breathing rate during the experiments.

Abdominal hair was removed using a commercially available chemical hair remover followed by application of ultrasound transmission gel (Aquasonic, Parker Laboratories, Fairfield, NY, USA). The ultrasound recordings were performed with a high-frequency ultrasound system (30 MHz, VisualSonics Vevo 770 system, Toronto, Canada) with an axial resolution of 55 *μ*m, a focal length of 12.7 mm, and a maximal field of view of 20 mm. Scanning time was kept as short as possible according to the ALARA principle [13]. Between the three subsequent developmental stages that were assessed the pregnant mice were housed in cages in the animal facility.

### 2.2. Ultrasound Recording Protocol

All ultrasound recordings were performed by NDH and EEC. During the ultrasound recordings the two laterally located uterus horns were scanned for the presence and position of the embryos/fetuses (*n* = 105). In individual embryos/fetuses, the left and right side were established to identify the position of the heart including the individual cardiac compartments and structures. At 12.5 dpc these compartments included the left atrium (LA), right atrium (RA), LV, RV, the common AV canal (cAVC) encompassing the future mitral valve (MV) and tricuspid valve (TV) orifices, common outflow tract (cOFT), developing interventricular septum (IVS), and the interventricular foramen, that is, the continuity between the LV and RV before completion of ventricular septation. At later stages (14.5 and 17.5 dpc) the MV and TV orifices, aorta (Ao), and pulmonary trunk (PT) were identified.

### 2.3. Pulsed-Wave Doppler Recordings

**Figure 1:**
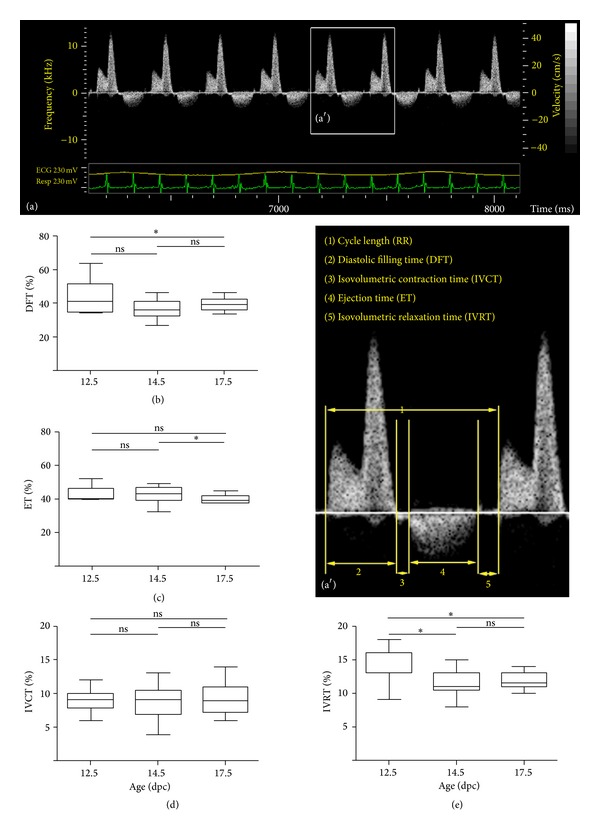
Development of LV inflow patterns. (a) Shows an example of a pulsed-wave Doppler recording across the MV at 17.5 dpc. (a′) Indicates the magnification of the boxed area in (a) in which the individual time intervals are indicated, that is, RR, DFT, IVCT, ET, and IVRT. (b) Through (e) indicates the course of the DFT, ET, IVCT, and IVRT, respectively, throughout embryonic/fetal life. All values in (b–e) are expressed as percentage of the RR.

The methods that were used for pulsed-wave Doppler flow recordings are summarized in Table 1. For flow measurements automatic Doppler angle corrections were accepted up to 45 degrees. Flow recordings across the cAVC (12.5 dpc: future MV *n* = 1; future TV *n* = 11) and developing MV and TV orifices (14.5 dpc: MV *n* = 17, TV *n* = 20; 17.5 dpc: MV *n* = 16, TV *n* = 14) were performed to assess the peak-E (early/passive ventricular filling by ventricular relaxation in early diastole) and peak-A wave velocities (active ventricular filling due to atrial contraction); hence the E/A ratios were calculated. The recordings across the MV orifice cAVC and developing were also used to evaluate the embryonic heart rate (HR) in beats per minute (BPM), cardiac cycle length (RR interval), diastolic ventricular filling time (DFT; 12.5 dpc, *n* = 11, 14.5 dpc, *n* = 17, and 17.5 dpc, *n* = 16), ejection time (ET; 12.5 dpc, *n* = 11, 14.5 dpc, *n* = 17, and 17.5 dpc, *n* = 16), isovolumetric contraction time (IVCT; 12.5 dpc, *n* = 7, 14.5 dpc, *n* = 17, and 17.5 dpc, *n* = 16), and isovolumetric relaxation time (IVRT; 12.5 dpc, *n* = 7, 14.5 dpc, *n* = 17, and 17.5 dpc, *n* = 16) of the LV (Figure 1(a)). The myocardial performance index (MPI; 12.5 dpc, *n* = 5; 14.5 dpc, *n* = 17; 17.5 dpc, *n* = 16), also known as Tei index, evaluates the combined LV systolic and diastolic function was calculated using the following formula: (IVRT + IVCT)/ET [14].

Pulsed-wave Doppler recordings were also performed in the cOFT at 12.5 dpc (*n* = 15) and Ao and PT at 14.5 dpc (*n* = 18 and *n* = 12) and 17.5 dpc (*n* = 12 and *n* = 4; Table 1). Furthermore, the velocity time integral (VTI), that is, the area under the Doppler velocity envelope, for one heart beat was determined (12.5 dpc, *n* = 15; 14.5 dpc, Ao, *n* = 18, and PT, *n* = 12; 17.5 dpc, Ao, *n* = 12, and PA, *n* = 4) and the diameters of the cOFT (*n* = 2) and Ao (14.5 dpc, *n* = 3, and 17.5 dpc, *n* = 2) were evaluated from B-mode echoloops in order to calculate the mean stroke volume (SV) per developmental stage according to the following formula: mean SV = mean TVI × (mean diameter of Ao or cOFT/2)^2^ × *π*. Hence, the mean cardiac output (CO) was calculated (mean CO = mean SV × mean HR) for the three developmental stages.

### 2.4. M-Mode Measurements

For morphological assessment M-mode measurements in short- or long-axis views of the ventricles were performed, to evaluate the end-systolic and end-diastolic ventricular inner diameter of the LV (LVID; 12.5 dpc, *n* = 9, 14.5 dpc, *n* = 11, and 17.5 dpc, *n* = 14) and RV (RVID; 12.5 dpc, *n* = 9, 14.5 dpc, *n* = 10, and 17.5 dpc, *n* = 12) and IVS diameter (12.5 dpc, *n* = 4, 14.5 dpc, *n* = 6, and 17.5 dpc, *n* = 9). Cardiac fractional shortening (FS%) for the LV and RV was calculated using the formula: (VIDdiastole − VIDsystole)/VIDdiastole × 100%. Due to the size of the hearts at 12.5 dpc, the resolution often limited the possibility to discriminate the epicardial, myocardial, and endocardial layer of the ventricular free wall. Therefore, at this stage the distances from the epicardial layer to the IVS in end-systole and end-diastole were used to calculate the FS%.

Although a complete hemodynamic and morphological assessment for each heart was aimed for (Table 2 for overview), imaging possibilities were regularly limited by the position of the embryo/fetus. All echocardiographic recordings were stored for offline data analysis by NDH and EEC; interobserved analysis demonstrated a high level of consistency (*r* = 0.8–1.0; *P* < 0.05). Flow parameters were calculated by the average of pulsed-wave Doppler complexes of three consecutive beats; similar methods were applied for M-mode recordings and diameter calculations on B-mode echoloops.

### 2.5. Cardiac Morphology and Three-Dimensional Reconstructions

After the experiments, the pregnant mouse was sacrificed by cervical dislocation and embryos/fetuses were extracted. We refer to previous publications for details regarding embryonic/fetal processing, sectioning, immunohistochemistry with MLC2a specific antibodies, and preparation of the three-dimensional AMIRA reconstructions (AMIRA software package, Template Graphics Software, San Diego, USA) [15].

### 2.6. Statistics

All statistics were performed with the statistical package for the social sciences 15.0 (SPSS Inc, Chicago, IL, USA) and GraphPad Prism (GraphPad Software, La Jolla, CA, USA). A *P* < 0.05 (2-tailed) was considered to be significant; all values are given as mean ± SEM. All boxplots indicate the upper and lower quartile of the median, which is indicated by the horizontal bar. The whiskers indicate max/min range of the values.

## 3. Results

### 3.1. Cardiac Morphology

Reliable imaging of hearts as small as 2 mm was feasible in all stages examined. Representative examples are shown in online Movie 1 (see Supplementary Material available online at http://dx.doi.org/10.1155/2014/531324).

**Figure 2:**
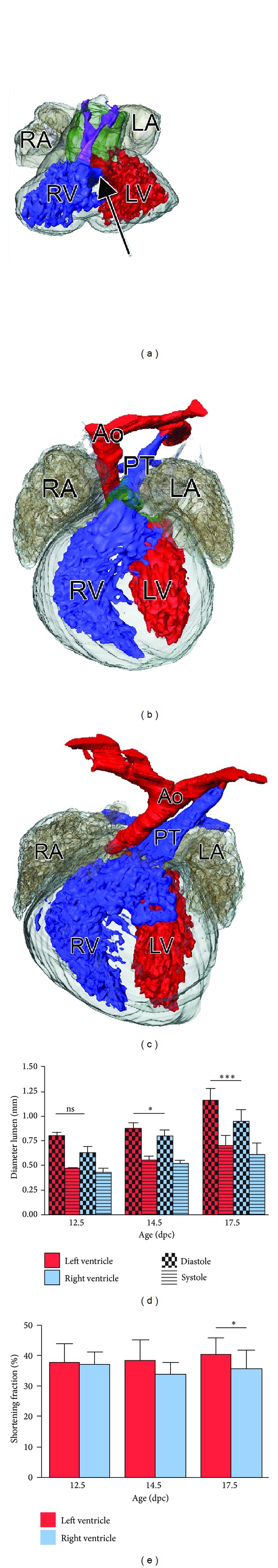
Cardiac morphology and FS% calculation. (a) Shows a reconstruction of the anterior view of an embryonic heart of 12.5 dpc. The myocardium is indicated in grey transparent. The LA and RA are indicated in transparent dark grey. The LV and RV lumen are indicated in red and blue, respectively. Note that the outflow tract lumen (purple) is positioned completely above the future RV, which is surrounded by large outflow tract cushions (green transparent). At these stages development of the IVS has not yet completed leading to a direct connection between the LV and RV via the interventricular foramen (arrow). (b) Anterior view of an early fetal heart of 14.5 dpc. At this stage IVS development has been completed and four separate cardiac chambers can be identified. The outflow tract consists of a separate Ao and PT including their valve apparatus, which at these stages mainly consist of cushion tissue (green transparent). (c) Anterior view of a late fetal heart of 17.5 dpc. At this stage the heart shows a mature morphological phenotype. (d) Schematic representation of LV and RV diameters and (e) FS% at the three consecutive stages of development.

At embryonic stages (12.5 dpc) ventricular septation has been initiated but is not completed resulting in a primitive interventricular foramen that directly connects the lumen of LV and RV. M-mode recordings at these stages show a mean systolic IVS diameter of 0.20 ± 0.045 mm.  Figure 2(a) shows a reconstruction of a 12.5 dpc embryonic heart, in which the lumen of the four cardiac chambers, interventricular foramen, and the OFT can be identified. The atrial and ventricular chambers are connected via the cAVC that harbors large AV cushions, which will contribute to formation of the MV and TV. The AV canal is still partly positioned above the LV at this stage. The developing OFT is still positioned completely above the RV and contains cushion tissue that will form the semilunar valves of the Ao and PT. They will further form the basis for the muscular subpulmonary infundibulum and will contribute to the membranous part of the IVS. Consequently, the blood from the LV runs via the primitive interventricular foramen into the cOFT, that is, future Ao and PT.

At early fetal stages of 14.5 dpc the heart has increased in size (Figure 2(b)) and ventricular septation is now complete. The systolic diameter of the IVS has increased (0.24 ± 0.018 mm). In the AVC the TV orifice has expanded and is now situated entirely above the RV. Also the MV orifice can be identified as well as the developing AV valves. The cardiac OFT now consists of a separate Ao and PT connecting to the LV and RV, respectively. The Ao and pulmonary valves still show immaturity and consist of cushion tissue.

At late fetal stages (17.5 dpc) the size of the RV and LV and IVS (0.39 ± 0.009 mm) has increased significantly (Figure 2(c)). Mature valves are present at the levels of the in- and outflow parts of both ventricles.

### 3.2. Diastolic and Systolic Function

During embryonic and fetal development the HR increases significantly (*P* < 0.0001) from 125 ± 9.5 up to 219 ± 8.3 BPM. Ventricular inflow patterns across the developing MV and TV annulus were studied by pulsed-wave Doppler imaging.

**Figure 3:**
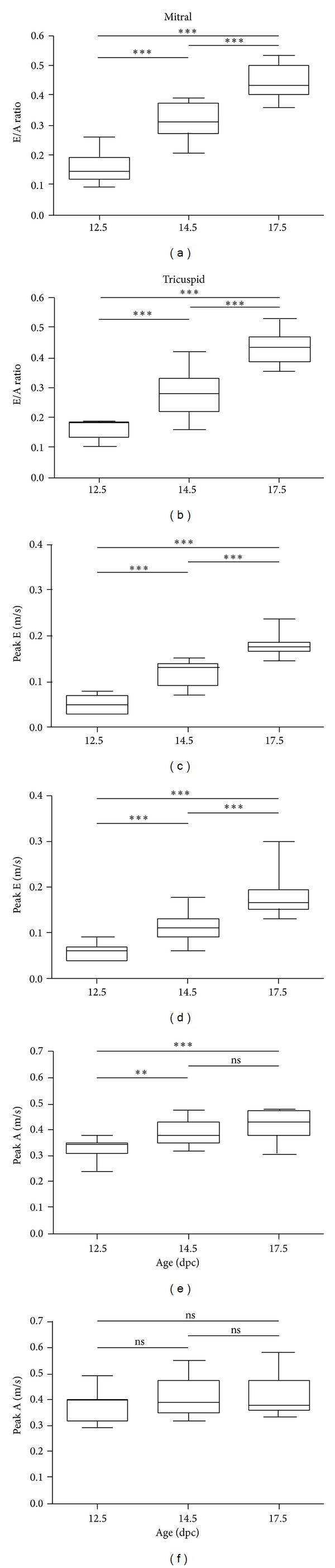
Development of LV and RV inflow patterns. The graphs represent the course of the E/A ratio (a, b), peak-E wave (c, d), and peak-A wave (e, f) across the developing MV and TV at the three subsequent developmental stages.

The DFT corrected for the RR interval (DFT/RR) did not differ significantly at the three stages assessed (Figure 1(b)). LV and RV filling were dominated by the peak-A wave. Between 12.5 and 17.5 dpc the peak-A wave of the developing MV annulus increased significantly (0.33 ± 0.001 m/s versus 0.42 ± 0.014 m/s; *P* < 0.0001) whereas that of the TV also showed an increasing trend, albeit not significant (0.37 ± 0.018 m/s versus 0.41 ± 0.019 m/s; *P* = NS). The peak-E wave also increased significantly for both LV (0.05 ± 0.006 m/s versus 0.18 ± 0.005 m/s; *P* < 0.0001) and RV (0.06 ± 0.006 m/s versus 0.18 ± 0.012 m/s; *P* < 0.0001) and showed a relatively greater increase than the peak-A wave during development. As a result, the E/A ratio, a measure for the ventricular diastolic function, showed a significant increase for both LV (0.16 ± 0.015 versus 0.44 ± 0.013; *P* < 0.0001) and RV (0.16 ± 0.010 versus 0.44 ± 0.014; *P* < 0.0001) (Figures 3(a)–3(f)).

The IVRT, one of the measures for diastolic LV function, corrected for the RR interval (IVRT/RR) demonstrated a significant (*P* = 0.037) decrease between 12.5 (0.15 ± 0.011) and 14.5 dpc (0.12 ± 0.004) and remained stable at later fetal stages 17.5 dpc (0.12 ± 0.003) (Figure 1(e)), indicating that LV myocardial relaxation remains stable after completion of IVS development during fetal life.

The systolic function of the developing ventricles was assessed through studying the course of the FS%, IVCT/RR, and ET/RR. Throughout embryonic and fetal development the FS% remained constant for both ventricles and values ranged between *≈*30 and 40%. Furthermore, except for 17.5 dpc (LV: 40.6%, RV 35.7%; *P* = 0.046) no significant differences were observed between the FS% of the LV and RV (Figures 2(d) and 2(e)). The mean IVCT/RR remained stable during all three stages *≈*0.09%; the ET/RR showed no changes between 12.5 dpc (0.42 ± 0.015) and 14.5 dpc (0.43 ± 0.010) but significantly decreased at 17.5 dpc (0.40 ± 0.006; *P* = 0.017) (Figures 1(c) and 1(d)).

A combined assessment of LV systolic and diastolic function was performed by calculating the MPI. At embryonic preseptated stages (12.5 dpc), the MPI was 0.64 ± 0.012 and decreased to 0.49 ± 0.017 (*P* = 0.0002) after closure of the IVS at 14.5 dpc. At late fetal stages (17.5 dpc) the MPI did not change significantly (0.53 ± 0.022; *P* = NS).

### 3.3. Outflow Tract and CO

**Figure 4:**
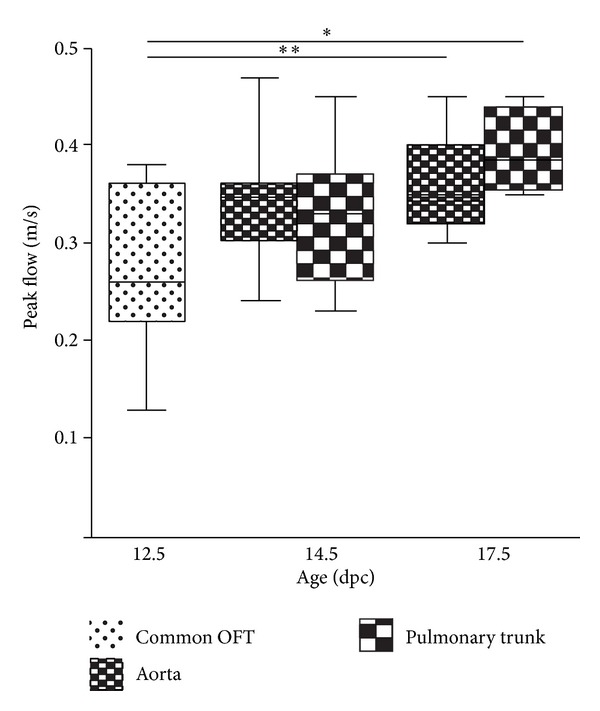
Pulsed-wave Doppler flow measurements in the cOFT, Ao, and PT. The graph demonstrates the significant increase of the peak blood flow measured in the cOFT at 12.5 and Ao and PT at 14.5 and 17.5 dpc.

The pulsed-wave Doppler recordings performed in the cOFT demonstrated a peak flow of 0.27 ± 0.021 m/s at preseptated stages. As from 14.5 dpc two individual ventricular outflow tracts could be discriminated; especially the high echodensity of the blood at these stages enabled to discriminate the flow in the Ao (0.34 ± 0.013 m/s) and PT (0.33 ± 0.021 m/s) that are closely related to each other. As can be observed in Figure 4, the peak flow increased significantly in the Ao (from 0.27 ± 0.021 m/s to 0.36 ± 0.016 m/s; *P* = 0.010) and PT (0.27 ± 0.021 m/s to 0.44 ± 0.030 m/s; *P* = 0.003) along fetal life.

Table 3 summarizes the HR, VTI, and the diameters of the cOFT and Ao that were used to calculate the mean CO at the three developmental stages. Between 12.5 dpc and 14.5 dpc the CO remained stable, where after it increased to 0.96 mL/min at 17.5 dpc.

## 4. Discussion

Heart development is a complex process, which has been extensively studied in both WT and genetically mutated mouse models [3]. It is only in the last decades that modern imaging techniques like ultrasound enable the assessment of real-time hemodynamic and morphological changes that occur during mouse heart development [4–6, 8, 16–18]. Knowledge regarding these functional parameters is essential since abnormal flow in the developing heart might lead to CHD through aberrant activation of shear-stress responsive genes important in cardiogenesis [9, 11]. In early reports describing the course of functional parameters during heart development major variability exists in measured values. This is most likely related to the relatively low ultrasound frequencies of clinical ultrasound machines used to image the small hearts of mouse embryos/fetuses [16] and externalization of uterus horns during ultrasound assessment [18], which influences embryonic/fetal physiology. Low dose isoflurane anesthesia, used in this study, only has a minimal effect on hemodynamics and diastolic function in adult mice and the effect on mouse embryos is supposed to be marginal [6].

In the current study we aimed to provide an overview of hemodynamic parameters and changes that occur at subsequent stages of heart development, using a high-frequency ultrasound system.

Compared to other reports in mouse using similar ultrasound techniques we did not only focus on development of the future LV but also on that of the RV. Key findings of our study are (1) a reliable assessment of LV and RV in- and outflow patterns can be performed from 12.5 dpc to term; (2) a significant improvement occurs in the diastolic and systolic function of both LV and RV; (3) over the developmental period of 12.5 dpc to 17.5 dpc, the ratio between RV and LV size, as well as the FS% of both ventricles, did not change significantly.

Knowledge regarding the functional parameters of the RV is important since the RV is commonly affected in CHD. During development, the RV including the TV annulus develops subsequent to the LV by addition of SHF derived cells to the developing heart [1]. Many genes have been identified that have an important role in the SHF contribution to the developing heart and mutations in these genes may lead to CHD [19].

At early embryonic stages endocardial cushion tissue is positioned in the primitive AVC and OFT, which later on will contribute to formation of the TV and MV and Ao and PT semilunar valves. Some clinical studies argued that AV valve regurgitation is a common phenomenon during the first trimester of pregnancy [20]. Our study, in accordance with several others in mouse [4, 6] and human [13] did not show signs of regurgitation at both early and late stages of heart development. Pulsed-wave Doppler recordings across the cAVC at 12.5 dpc and MV and TV at 14.5 dpc demonstrated that the AV cushions mimic the function of normal AV valves at 17.5 dpc. In our opinion especially the flow in the cOFT at early embryonic stages, which is completely positioned above the RV (see Figure 2(a)), might wrongly be identified as regurgitation of the developing AV valves/cushions. This stresses the importance of detailed knowledge regarding the rapid morphological changes that the heart undergoes at especially the very early developmental stages.

Regarding growth of the heart we demonstrated that there was a 31% increase of LVID, 34% of RIVD, and 35% of IVS diameter from 12.5 up to 17.5 dpc. We showed a trend towards a smaller RVID as compared to the LVID at all stages, which was not mentioned in other studies.

### 4.1. Diastolic and Systolic Function during Heart Development

Improvement of ventricular systolic and diastolic function occurs throughout the length of embryonic and fetal development. With respect to the diastolic function we demonstrated a significant increase of E/A ratio across the developing MV and TV orifices, which was related to an increase of the peak-E wave, that is, the fraction of diastolic ventricular filling that is defined by ventricular relaxation. These data are in accordance with the study of Zhou and colleagues, who demonstrated that the E/A ratio is increasing during development, which seems to continue up to approximately 3 weeks after birth [6]. Around day four after birth, the peak-E wave measured across the MV becomes more dominant than the peak-A wave indicating an improvement of cardiac compliance and that LV filling becomes dominated by passive ventricular relaxation. Remarkably, these changes were not observed for the RV inflow patterns, where the peak-A wave remains dominant [6]. Human embryonic and fetal hearts show the same maturation course of E/A ratios across the developing MV and TV as was demonstrated in multiple studies [21–25].An abnormal course of E/A ratio, that is, a decreasing [25, 26] or an increasing trend [27] along development, has been related to the presence of CHD.

With respect to systolic ventricular function, the FS% remained stable (*≈*30–40%) for both ventricles throughout development. We did find a significant difference favoring the LV FS% as compared to the RV FS% at 17.5 dpc. Interestingly, the same phenomenon of FS% difference between LV and RV has been reported in a study of human fetal hearts between 14 and 40 weeks of pregnancy. However, as in mouse, differences between human LV and RV FS% is subtle, [28] and whether a decreased or decreasing FS% is a predictor of CHD remains unknown. Furthermore, we did not find a decreasing trend of both LV and RV FS% as was recently demonstrated during human fetal life [28, 29]. With respect to the CO we noticed no change around the proces of ventricular septation (between 12.5 and 14.5 dpc), where after a major increase occured to 0.96 mL/min near term (17.5 dpc). The initial CO decrease might be related to the fact that the mean diameter of the unseptated cOFT diameter was used at 12.5 dpc compared to the isolated Ao diameter at 14.5 dpc. The subsequent CO increase is mostly related to the dramatic increase of HR along fetal life.

The MPI or Tei index [14] is a measure for combined systolic and diastolic cardiac function and can be calculated for both ventricles. In order to assess the MPI three different time intervals are needed, the IVCT, IVRT, and ET. For the LV these parameters can be easily obtained, since all the three parameters can be assessed at once by placing the Doppler ultrasound beam across the developing MV orifice. Noticeably, in literature there is less data regarding RV MPI and its course along development, which might be related to the fact that for the RV not all the three time intervals can be assessed in one view as was also suggested in a recent review by Godfrey et al. [14]. The current study demonstrated a significant decrease of LV MPI between 12.5 and 14.5 dpc, which might be related to the rapid morphological changes the heart goes through including completion of IVS leading to altered hemodynamic conditions in the developing heart. It is however also important to notice that at preseptation stages the IVCT and IVRT cannot always be identified separately so that calculation of the MPI is frequently not possible at very early stages of embryonic development. Between 14.5 and 17.5 dpc we noticed a slight increase of the MPI suggesting that cardiac function improves at subsequent fetal stages; similar results have also been demonstrated in mouse [4] and human [30]. However, discussion remains regarding the exact course of the MPI, IVCT, IVRT, and ET and whether isolated changes of one of the factors is a predictor for fetal demise and CHD. Interestingly with respect to IVRT% we observed a decreasing trend near completion of the IVS (between 12.5 and 14.5) whereafter it more or less remained stable throughout fetal life. A similar course was observed in a recent ultrasound study in embryonic/fetal C57Bl6 mice [4]. Human embryos also showed a decreasing course of the IVRT% [13, 21], and an elevated IVRT% has been implicated as a predictor of CHD [13].

## 5. Conclusion

We demonstrated the course of several morphologic and hemodynamic parameters along embryonic and fetal life. Our data show that a reliable assessment can be made not only of the left, but also of right ventricular function during embryonic/fetal stages. We demonstrate an overall improvement of cardiac diastolic and systolic function of both LV and RV along heart development. The implementation of high-frequency ultrasound in embryonic/fetal mouse heart development is a promising and useful tool. The data presented in this study might be useful as reference values in future studies in genetic mutated mouse models of CHD.

## Supplementary Material

Representative B-mode movies of 4-chambers view at 12.5, 14.5 and 17.5 dpc. The LA, LV, RA and RV can be discriminated.

## Figures and Tables

**Table 1:** The methods used for performing Doppler flow recordings per gestational age.

Age (dpc) and alignment	Location	Parameter (unit)	View	Doppler beam: position
12.5	Future MV	E-wave (m/s) A-wave (m/s) DFT (ms) IVCT (ms) IVRT (ms) ET (ms)	4 chambers	cAVC, parallel to flow direction at left side of the developing IVS
	Future TV	E-wave (m/s) A-wave (m/s)	4 chambers	cAVC, parallel to flow direction at the right side of the developing IVS
	cOFT, future Ao/PT	Peak flow (m/s) VTI (mm)	5 chambers/OFT	Proximal part cOFT, parallel to flow direction

14.5 and 17.5	MV	E-wave (m/s) A-wave (m/s) DFT (ms) IVCT (ms) IVRT (ms) ET (ms)	4 chambers	LV below (developing) MV annulus, parallel to flow direction at left side of the IVS
	TV	E-wave (m/s) A-wave (m/s)	4 chambers	RV below (developing) TV annulus, parallel to flow direction at right side of IVS
	Ao	Peak flow (m/s)	5 chambers/LVOT	Ao above (developing) valve annulus, VTI (mm) parallel to flow direction
	PT	Peak flow (m/s)	RVOT	PT above (developing) valve annulus, parallel to flow direction

**Table 2:** Summary of the assessment of embryonic and fetal hearts.

Method	Parameter	Unit	Structure (abbreviation)	
2D ultrasound B-mode echoloops	Morphological assessment, position, and diameters of cardiac compartments.	mm	Right atrium (RA), left atrium (LA), right ventricle (RV), left ventrile (LV), common outflow tract (cOFT), pulmonary trunk (PT), aorta (Ao), inter ventricular septum (IVS), common atrioventricular canal (cAVC), mitral valve (MV), and tricuspid valve (TV)	

			Definition	Formula

Pulsed-wave Doppler	Peak E	m/s	Early, diastolic ventricular filling	
	Peak A	m/s	Active, diastolic ventricular filling	
	E/A ratio			
	RR interval	ms	Cycle length/evaluation of heart rate	
	DFT	ms	Diastolic filling time	
	IVRT	ms	Isovolumetric relaxation time	
	IVCT	ms	Isovolumetric contraction time	
	ET	ms	Ejection time	
	MPI/Tei index		Myocardial performance index	IVRT + IVCT/ET
	Peak velocities cOFT, Ao, PT	m/s		
	VTI	mm	Velocity time integral	
	SV		Stroke volume	SV = TVI × (Ao or cOFT diameter/2)^2^ × *π*
	CO	mL/min	Cardiac output	
M-mode	VID	mm	Ventricular inner diameter	
	LVIDd	mm	End diastolic left ventricular inner diameter	
	LVIDs	mm	End systolic left ventricular inner diameter	
	RVIDd	mm	End diastolic right ventricular inner diameter	
	RVIDs	mm	End systolic right ventricular inner diameter	
	IVSd	mm	End diastolic interventricular septum diameter	
	IVSs	mm	End systolic interventricular septum diameter	
	FS	%	Fractional shortening	(VIDd − VIDs)/VIDd × 100%

**Table 3:** The parameters used for calculation of the mean CO per gestational age.

Parameter	12.5 dpc (cOFT)	14.5 dpc (Ao)	17.5 dpc (Ao)
VTI (mm)	33.9 ± 1.37	42.3 ± 2.0	29.2 ± 2.05
Mean diameter (mm)	0.23	0.18	0.48 ± 0.03
Mean HR (bpm)	134 ± 11.2	148 ± 6.1	206 ± 9.8
Mean CO (mL/min)	0.19	0.16	0.96
